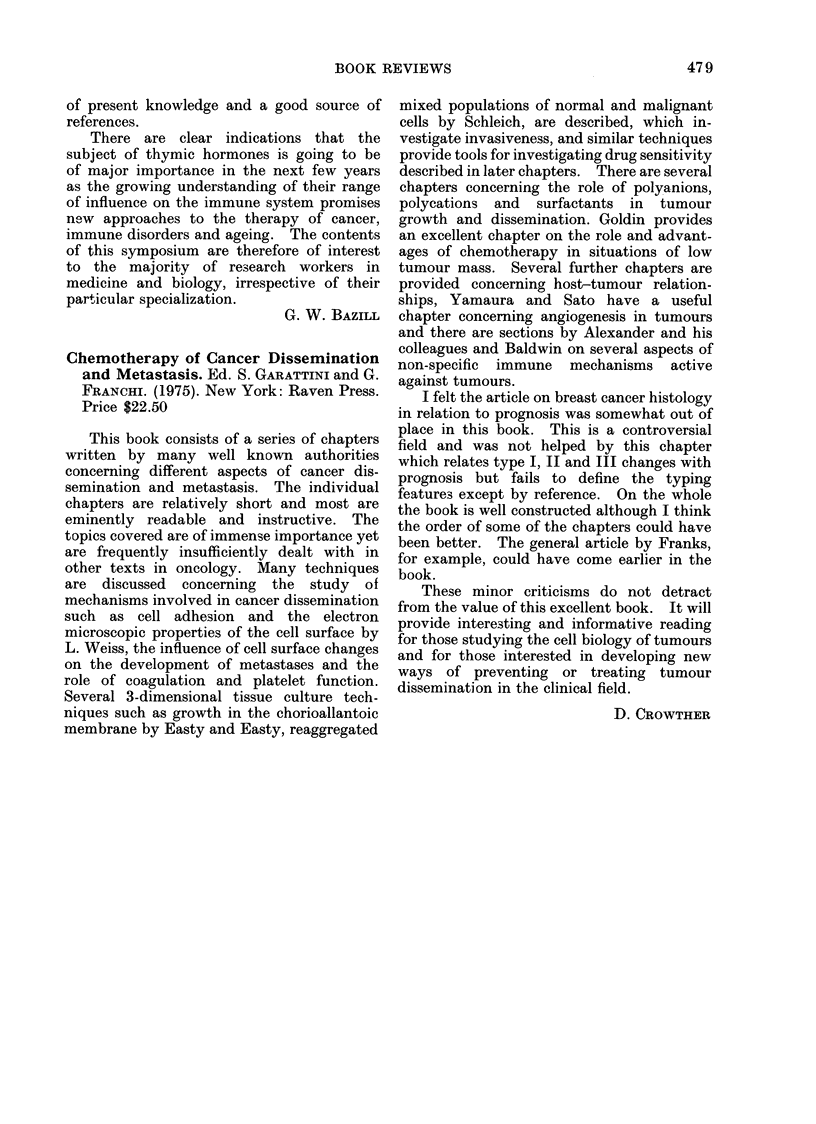# Chemotherapy of Cancer Dissemination and Metastasis

**Published:** 1976-04

**Authors:** D. Crowther


**Chemotherapy of Cancer Dissemination and Metastasis.** Ed. S. GARATTINI and G. FRANCHI. (1975). New York: Raven Press. Price $22.50

This book consists of a series of chapters written by many well known authorities concerning different aspects of cancer dissemination and metastasis. The individual chapters are relatively short and most are eminently readable and instructive. The topics covered are of immense importance yet are frequently insufficiently dealt with in other texts in oncology. Many techniques are discussed concerning the study of mechanisms involved in cancer dissemination such as cell adhesion and the electron microscopic properties of the cell surface by L. Weiss, the influence of cell surface changes on the development of metastases and the role of coagulation and platelet function. Several 3-dimensional tissue culture techniques such as growth in the chorioallantoic membrane by Easty and Easty, reaggregated mixed populations of normal and malignant cells by Schleich, are described, which investigate invasiveness, and similar techniques provide tools for investigating drug sensitivity described in later chapters. There are several chapters concerning the role of polyanions, polycations and surfactants in tumour growth and dissemination. Goldin provides an excellent chapter on the role and advantages of chemotherapy in situations of low tumour mass. Several further chapters are provided concerning host–tumour relationships, Yamaura and Sato have a useful chapter concerning angiogenesis in tumours and there are sections by Alexander and his colleagues and Baldwin on several aspects of non-specific immune mechanisms active against tumours.

I felt the article on breast cancer histology in relation to prognosis was somewhat out of place in this book. This is a controversial field and was not helped by this chapter which relates type I, II and III changes with prognosis but fails to define the typing features except by reference. On the whole the book is well constructed although I think the order of some of the chapters could have been better. The general article by Franks, for example, could have come earlier in the book.

These minor criticisms do not detract from the value of this excellent book. It will provide interesting and informative reading for those studying the cell biology of tumours and for those interested in developing new ways of preventing or treating tumour dissemination in the clinical field.

D. CROWTHER